# Impact of Ceramide Acyl Chain Length on Human Skin Barrier Recovery and Hydration

**DOI:** 10.1111/jocd.70795

**Published:** 2026-03-11

**Authors:** Do‐Hyeon Gwon, Hyun Kyung Choi, Eun Ok Lee, Sung Kyu Hong, Chang Seo Park, Jin Wook Kim, Kwang‐Hyeon Liu

**Affiliations:** ^1^ BK21 FOUR KNU Community‐Based Intelligent Novel Drug Discovery Education Unit Research Institute of Pharmaceutical Sciences, College of Pharmacy, Kyungpook National University Daegu Republic of Korea; ^2^ Department of Chemical and Biochemical Engineering Dongguk University Seoul Republic of Korea; ^3^ LCS Biotech Yongin Republic of Korea

**Keywords:** ceramide, chemical analysis, cohesion, hydration, skin barrier

## Abstract

**Objective:**

To compare the effects of ceramide acyl chain length on human skin barrier function.

**Methods:**

Mixtures of phytoceramide containing non‐hydroxy fatty acids (CER NPs) with different acyl chain lengths and corresponding test creams were prepared: C16–C24 CER NP and C24–C30 CER NP (ultra‐long‐chain, ULC CER NP). The content of C24 CER NP in these formulations was 0.3% and 39%, respectively. Liquid chromatography‐tandem mass spectrometric (LC–MS/MS) analysis of skin ceramides was performed using tape‐stripped human stratum corneum (SC) samples. A vehicle‐controlled intra‐subject human study was conducted to assess acute skin barrier recovery, skin hydration, and SC cohesion.

**Results:**

Levels of C24 and C26 ceramides were significantly increased in skin treated with C24‐C30 CER NP. These analytical results were consistent with the findings from the human efficacy study. Functional evaluations demonstrated that C24–C30 CER NP significantly enhanced barrier recovery, skin hydration, and SC cohesion compared with C18 CER NP, whereas the C16–C24 CER NP formulation primarily improved skin hydration. Overall, the C24–C30 CER NP formulation exhibited the strongest barrier‐enhancing effects among the tested formulations.

**Conclusion:**

This study provides the first in vivo human evidence that ceramides with longer acyl chains confer superior improvements in skin barrier function compared with shorter‐chain ceramides. These findings highlight the critical role of acyl chain length in ceramide‐mediated barrier enhancement and support the rational design of ceramide‐based formulations for optimized skin barrier restoration.

## 1 Introduction

Ceramides (CERs) are essential lipid components of the human stratum corneum (SC), where they act synergistically with cholesterol and free fatty acids (FFAs) to establish and maintain the skin's permeability barrier [1, 2, 3, 4, 5]. The barrier properties of the epidermis are strongly dependent on both the composition and concentration of CERs. Structurally, each CER molecule consists of a long‐chain base (LCB) linked to a fatty acid (FA) via an amide bond. Structural variability arises from differences in carbon chain length, degree of unsaturation, and the presence and position of hydroxyl groups on both the LCB and FA moieties [6]. The human SC contains four principal types of LCBs—dihydrosphingosine (DS) or sphinganine (G), sphingosine (S), phytosphingosine (PS), and 6‐hydroxy sphingosine (H)—and three FA types: non‐hydroxy FA (N), α‐hydroxy FA (A), and esterified ω‐hydroxy FA (EO) [7]. The combinatorial pairing of these structural elements yields at least 12 distinct CER subclasses. Recently, 1‐*O*‐acylceramide, a novel CER subclass, has been identified in human skin [8, 9, 10]. Among these, CER ENP, a PS‐based 1‐*O*‐acylceramide, has been shown to synergistically enhance the barrier function of phytoceramide containing non‐hydroxy fatty acids (CER NP) when applied in specific ratios [11, 12, 13]. Alterations in the relative concentration of CER subclasses can impair barrier function. For example, individuals with atopic dermatitis commonly exhibit a marked reduction in the non‐hydroxy acyl phytosphingosine (NP) to non‐hydroxy acyl sphingosine (NS) ratio, which typically decreases from a normal value of approximately 3:1 [14, 15]. A similar imbalance is also observed in the NP‐to‐α‐hydroxy acyl phytosphingosine (AP) ratio. Prior studies have demonstrated that maintaining an NP‐to‐AP ratio of 2:1 is critical for sustaining regular lamellar lipid organization within the SC [16]. These findings underscore the critical role of CER subclass balance and, more broadly, CER molecular diversity in preserving the structural and functional integrity of the skin barrier.

The significance of CER chain length in skin barrier function was first highlighted by Wertz et al. in 1985, who analyzed the profile of amide‐linked FAs within the human SC. Their findings revealed that C24 and C26 were the most abundant FAs in several CER subclasses, whereas C30 was predominantly associated with EO‐type CERs [17]. Since that pioneering study, research has consistently confirmed the dominance of ultra‐long‐chain (ULC) FAs—particularly C24, C26, and longer—in most SC CER classes. Subsequent lipidomic analyses have shown that CERs with C26 and C28 FA moieties are especially prevalent in CER NP, CER NH, CER AP, and CER AH, whereas CER AS contains primarily shorter chains such as C16. The ω‐esterified CER subclasses, including CER EOS, CER EOP, and CER EOH, incorporate FAs exceeding C30 [18]. More recent comprehensive profiling further supports these findings, indicating that approximately 80%–90% of SC CERs contain FAs longer than C24 [19]. Among these, C30 has been identified as the predominant species within the EO‐type CERs. Collectively, these data underscore the central role of ULC CERs in shaping the biophysical properties of the SC lipid matrix and suggest that chain length is a key determinant of CER function in skin barrier homeostasis. Variations in CER chain length have been associated with differences in skin barrier function across both physiological and pathological conditions. In particular, studies have demonstrated that CERs extracted from atopic dermatitis lesions exhibit shorter average chain lengths compared with those from healthy skin, suggesting a strong correlation between CER chain length and skin barrier integrity [20].

Notably, variations in CER composition and chain length are not restricted to diseased states. Site‐specific differences in the skin have been documented, with distinct anatomical regions (e.g., face, leg, scalp, and lip) exhibiting unique CER profiles that correlate with skin‐site‐specific barrier properties [21]. Furthermore, disparities in CER chain length have been identified among healthy individuals, distinguishing between dry and non‐dry skin phenotypes. Recent evidence suggests that levels of long‐chain CERs, particularly C26 and C28, are markedly reduced in dry skin compared with non‐dry skin [22]. Collectively, these findings highlight the critical role of CER chain length in maintaining skin barrier function and suggest that deviations in chain length profiles may serve as biomarkers for barrier dysfunction across a range of conditions [23].

The relationship between CER chain length and skin barrier function has also been explored in simplified in vitro model membrane systems. Models incorporating well‐defined CERs, such as C16 and C24, have provided critical insights into the biophysical mechanisms underlying barrier regulation [24]. Notably, an increased proportion of C24 relative to C16 promotes the formation of orthorhombic lamellar phases, which are highly ordered lipid structures associated with enhanced barrier integrity and reduced permeability [25]. Complementary findings from molecular dynamics simulations further support the influence of chain length on membrane architecture. Simulated bilayers composed of CERs with varying chain lengths demonstrate that bilayer thickness increases with chain length [26]. Moreover, membranes containing shorter‐chain CERs exhibit higher permeability by an order of magnitude compared with those with longer‐chain CERs [27]. Experimental and computational evidence converge to show that the CER chain length governs lipid organization and barrier performance, elucidating the molecular basis for its role in skin barrier homeostasis [23, 28].

Since their identification as major SC components in 1985, CERs have been extensively incorporated in cosmetic formulations for more than 3 decades. Their use has expanded beyond traditional skincare to include lip care, hair care, and dermocosmetics. Among commercially available CERs, CER NP—introduced in 1994—was the first to be marketed as “skin‐identical,” owing to its *D*‐erythro stereochemical configuration that mirrors endogenous human CERs. However, its FA chain is restricted to C18, a limitation compared with the ULC CERs typically found in human skin. Although more than 20 structurally distinct CER classes have been identified in the SC, current cosmetic formulations generally incorporate only 9–10 of these classes. Furthermore, most commercial CERs do not replicate the full structural complexity of native CERs, particularly in terms of FA chain length. The majority of widely used synthetic CERs are based on shorter chains (e.g., C18), despite evidence that functional skin barrier formation requires CERs with chain lengths exceeding C24.

To date, no studies have directly compared the effects of ULC CERs with those of shorter‐chain analogs in human skin applications. In this context, the present study provides novel in vivo evidence that a topical formulation containing ULC CER, cholesterol, and free FAs significantly enhances multiple SC parameters. These improvements include accelerated acute barrier recovery, increased skin hydration, and enhanced SC cohesion, underscoring the superior efficacy of ULC CERs in restoring and maintaining skin barrier function.

## 2 Materials and Methods

### 2.1 Ceramides and Test Creams

Ceramide NP (CER) was prepared using phytosphingosine (PS) as the sphingoid long chain base (LCB) and fatty acid (FA) pools ranging from C16 to C30. Two CER mixtures were prepared according to previously published protocols with modifications [29]: one containing CERs with FA chains from C16 to C24, and another enriched with ULC CERs containing FA chains from C24 to C30. The chain lengths of *N*‐acylated FAs were confirmed using gas chromatography (GC). The chain length distribution of the prepared ULC CERs is summarized in Table 1. The composition of the C16–24 CERs corresponds to that of HP EcoCeramide LCS^R^ (LCS Biotech) and can be referenced in the associated formulation publication. C18 Cer NP (S2) obtained from Croda Korea served as the positive control, while S1 was the base cream containing no CERs or sphingoids. For the human study, 0.5% (*w/w*) of each CER was incorporated into a base cream.

**TABLE 1:** Chain length profiles of *N*‐acylated fatty acids in the respective samples (S1, base cream; S2, C18 CER; S3, C16–24 CER; and S4, C24–30 CER).

Cream sample	Chain length profile of *N*‐acylated fatty acids (%)							
	C16	C18	C20	C22	C24	C26	C28	C30
**S1**	—	—	—	—	—	—	—	—
**S2**	—	100	—	—	—	—	—	—
**S3**	4.9	86.0	5.9	2.9	0.3	—	—	—
**S4**	—	—	—	—	39.0	19.0	32.0	10.0

### 2.2 Human Subjects

A double‐blind, intra‐subject, vehicle‐controlled human study was conducted to evaluate the effects of CERs with varying acyl chain lengths on skin barrier parameters. Twenty‐one healthy young women (mean age 24.7 ± 4.7 years) with no history of dermatological disorders were recruited, and written informed consent was obtained from all participants. The test sites were limited to the inner areas of the left and right forearms. Participants were acclimatized for 30 min under controlled environmental conditions (temperature: 21.6°C ± 2.2°C, humidity: 48.0% ± 6.5%) before measurements. Skin barrier parameters evaluated include acute barrier recovery rate, SC hydration, and SC cohesion.

### 2.3 Liquid Chromatography‐Tandem Mass Spectrometric (LC–MS/MS) Analysis of Ceramides From Human SC Samples

SC samples were collected using D‐squame tape (22 mm diameter, CuDerm, Dallas, TX, USA) from the forearm skin of participants who provided informed consent. Six consecutive D‐squame strips were collected from each subject. the first and second strips were discarded, and the remaining four strips were stored separately. CER extraction from D‐squame tapes was performed using a previously described method [30]. Dried extracts were reconstituted in 100 μL of a chloroform/methanol mixture (1:9, *v/v*) containing an internal standard (IS; CER NP d18:1/16:0‐d₃₁, Avanti Polar Lipids, Alabaster, AL, USA), and analyzed using a triple quadrupole LC–MS/MS. Skin CERs were analyzed using a Nexera 2 liquid chromatographic system coupled to an LC–MS 8060 triple quadrupole mass spectrometer (Shimadzu Corporation, Kyoto, Japan) according to previously described methods [31, 32]. CER NP species were quantified by selected reaction monitoring (SRM) of the [M + H]^+^ precursor ion and the corresponding product ions. Each CER NP species was quantified as the peak area ratio of the target analyte to the IS, multiplied by the known concentration of the IS [6].

### 2.4 Measurement of Skin Barrier Recovery Rate, Hydration, and SC Cohesion

Acute barrier recovery was measured as previously described with slight modifications [13]. In the case of D‐squame tape‐induced damage, serial tape stripping was applied to a 2.5 cm^2^ area on the forearm skin until the transepidermal water loss (TEWL) reached 35–40 g h^−1^ m^−2^. TEWL was measured using a Tewameter MPA 580 (Courage & Khazaka, Cologne, Germany). Three consecutive measurements were averaged for each site. Barrier recovery rate was expressed as the percentage of TEWL relative to the untreated skin site (baseline).

SC hydration was assessed as previously described [29]. Samples were applied to designated sites on the forearm skin for 28 days. Hydration measurements were performed using a Corneometer CM820 device (Courage & Khazaka, Cologne, Germany), and skin hydration after 4 weeks of application was expressed in arbitrary units (AU) based on electrical capacitance.

SC cohesion, defined as the integrity of the SC in maintaining its structure against external physical stress, was evaluated after 4 weeks of sample application. Physical stress was applied via tape stripping, and SC cohesion was assessed by comparing TEWL values measured before and after 15 consecutive tape stripping repetitions [33].

### 2.5 Statistical Analysis

Data are presented as mean ± standard deviation (SD). Differences between means were assessed using one‐way analysis of variance (ANOVA) followed by Tukey's honest significant difference test (HSD), performed with IBM SPSS Statistics for Windows, version 23 (IBM Corp., Armonk, NY, USA). Statistical Significance was defined as a *p* < 0.05.

## 3 Results

### 3.1 Ceramide Profiling

**FIGURE 1:**
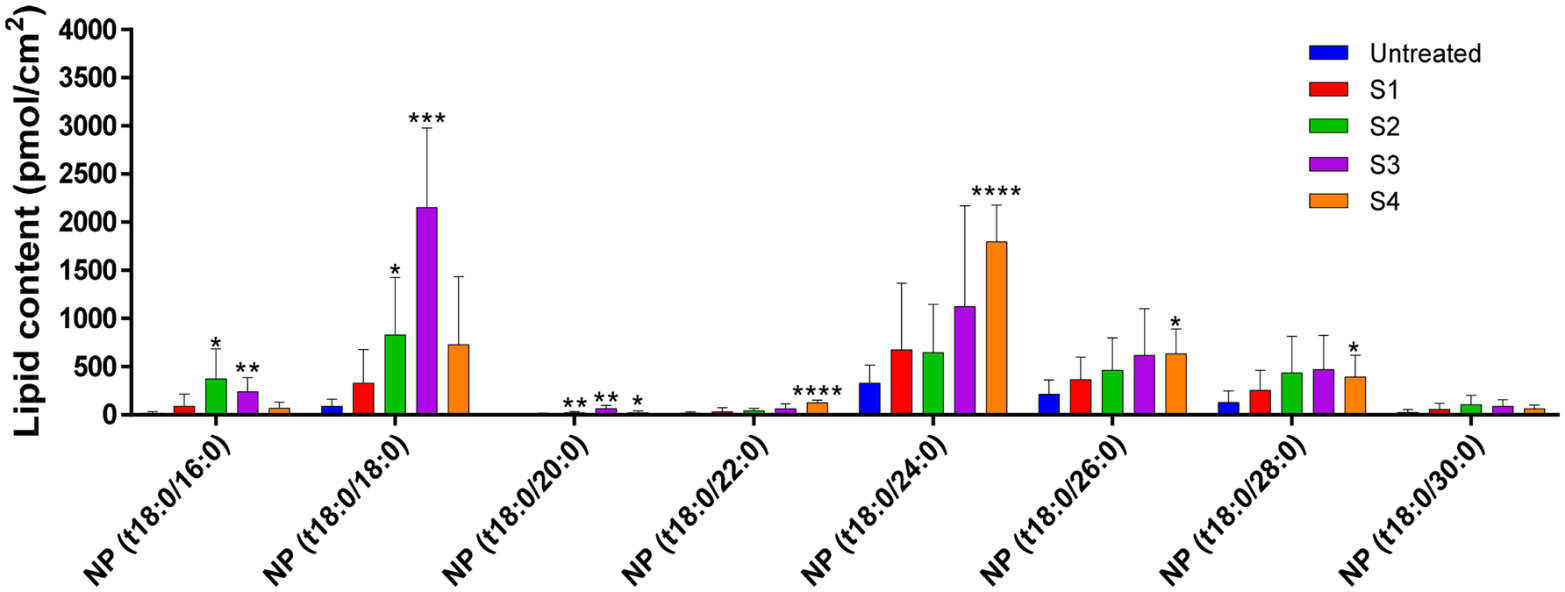
Effects of the test creams on NP‐type CER levels in human SC. SC samples were collected from the inner forearm following twice‐daily application of each test cream (S1, base cream; S2, C18 CER; S3, C16–24 CER; and S4, C24–30 CER) for 4 weeks. The first and second tape strips were discarded, and the third through sixth strips were used for CER analysis. Lipid content was quantified as NP‐type CER levels (pmol/cm^2^) and is presented as mean ± SD (*n* = 3). Statistical significance was determined using an unpaired t‐test, with comparisons made to the untreated group (*p < 0.05, **p < 0.01, ***p < 0.001, ****p < 0.0001).

In human SC, eight NP‐type CER NP species were analyzed. Levels of CER NP species containing C16:0, C18:0, and C20:0 acyl chains were significantly higher (*p* < 0.05) in epidermis treated with S2 and S3 compared with untreated groups. Similarly, levels of CER NP species containing acyl chains of C24–C30 were significantly elevated (*p* < 0.05) in epidermis treated with S4 compared with untreated skin (Figure 1).

### 3.2 Measurement of Barrier Recovery Rate

**FIGURE 2:**
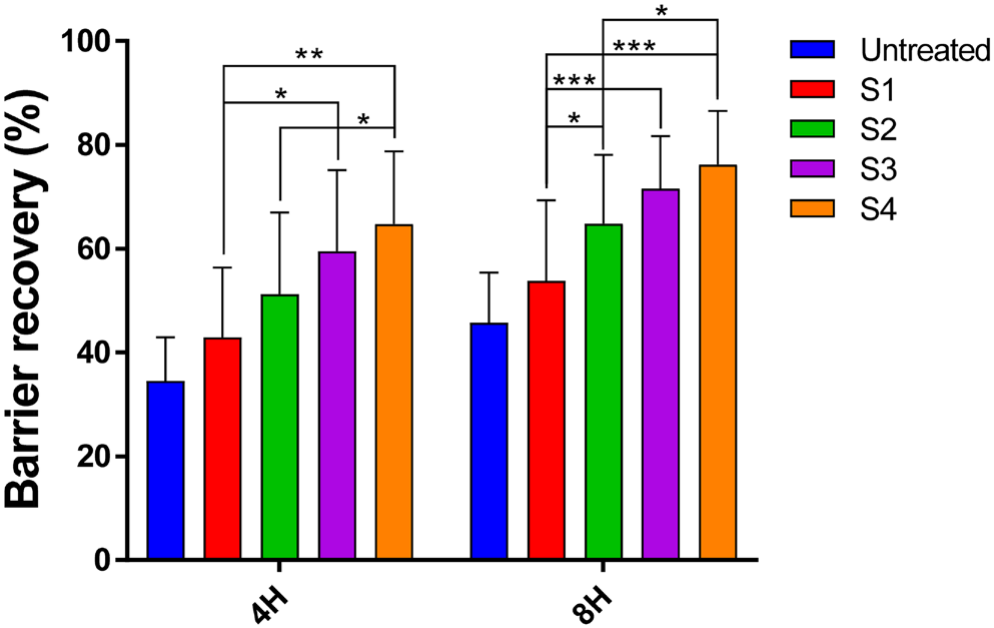
Effects of the test creams on skin barrier recovery in human participants. TEWL was measured 4 h (4H) and 8 h (8H) after application of the test creams (S1, base cream; S2, C18 CER; S3, C16–24 CER; and S4, C24–30 CER). Data were analyzed using one‐way ANOVA followed by Tukey's HSD test with IBM SPSS Statistics for Windows, version 23 (IBM Corp., Armonk, NY, USA). Statistical significance was defined as *p* < 0.05 (*p < 0.05, **p < 0.01, ***p < 0.001). Results are presented as mean ± SD (*n* = 20).

Skin barrier disruption was induced on each participant's forearm via tape stripping until the TEWL exceeded 30 g h^−1^ m^−2^. The base cream and test formulations containing 0.5% C18 CER, C16–24 CER, C24 CER, or C16–30 CER were immediately applied. Barrier recovery was assessed using a Tewameter at 4 and 8 h post‐application (Figure 2). One of the participants was disqualified for failing to complete the scheduled measurement. Compared with the vehicle control, all CER‐containing formulations (C18 CER, C16–24 CER, and C24–30 CER) improved barrier recovery at 8 h, with C24–30 CER showing the most rapid effect in a chain length–dependent manner. The test cream containing C24–30 CER achieved 76% recovery at 8 h, significantly exceeding the 65% observed with the positive control C18 CER. However, C16‐24 CER did not show statistically significant recovery than C18 CER. These findings indicate a strong correlation between ceramide acyl chain length and the rate of damaged skin barrier restoration; in other words, the longer the chain length, the faster the recovery.

### 3.3 Measurement of Skin Hydration

**FIGURE 3:**
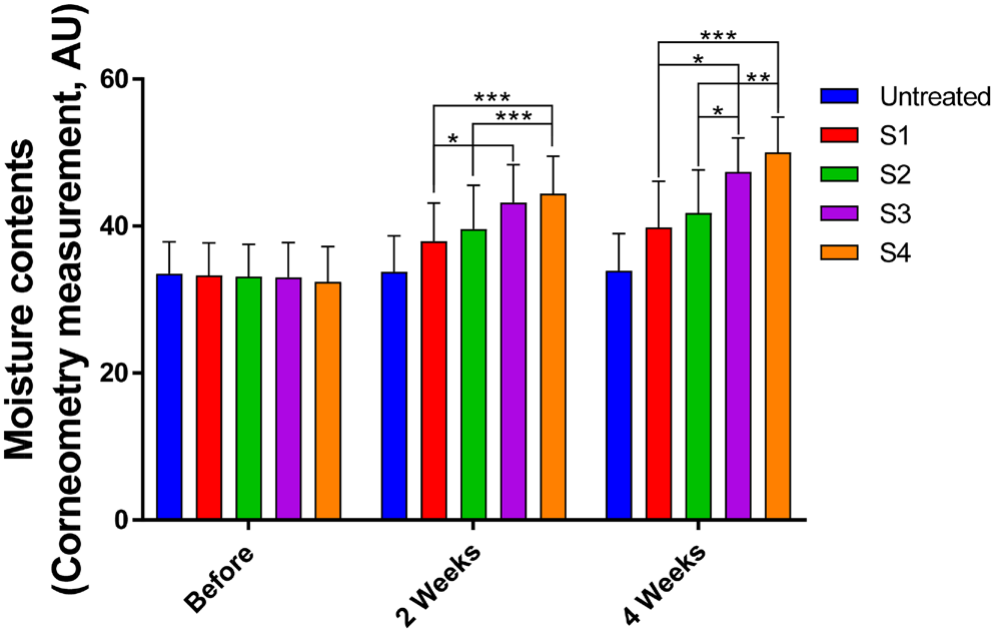
Effects of the test creams on skin hydration in human participants. Hydration was measured after 2 and 4 weeks of application of the test creams (S1, vehicle control; S2, C18 CER; S3, C16–24 CER; and S4, C24–30 CER). Values were expressed in AU based on capacitance values obtained using a Corneometer. Data were analyzed using one‐way ANOVA followed by Tukey's HSD test. Statistical significance was defined as *p* < 0.05 (*p < 0.05, **p < 0.01, ***p < 0.001). Results are presented as mean ± SD (*n* = 21).

Participants applied a fixed amount of each test formulation to a designated area on the forearm twice daily—once in the morning and once in the evening. Skin hydration was assessed using a Corneometer at week 2 and at the end of the 4‐week study. All 21 participants completed the trial. At week 2, hydration levels increased by 19% with C18 CER, 29.3% with C16–24 CER, and 31.7% with C24–30 CER relative to the untreated control. By week 4, these values further increased to 21.6%, 39%, and 47%, respectively. Direct comparisons showed that both C16–24 CER (*p* < 0.05) and C24–30 CER (*p* < 0.01) provided significantly greater hydration than C18 CER after 4 weeks of application, confirming the superior moisturizing efficacy of ULC CERs (Figure 3). No significant difference was observed between the C16–24 CER and C24–30 CER groups; however, when compared with C18 CER, only C24–30 CER demonstrated a significantly higher hydration level at week 2. Overall, C24–30 CER consistently produced the greatest improvement in skin hydration throughout the study.

### 3.4 Measurement of Skin Cohesion

**FIGURE 4:**
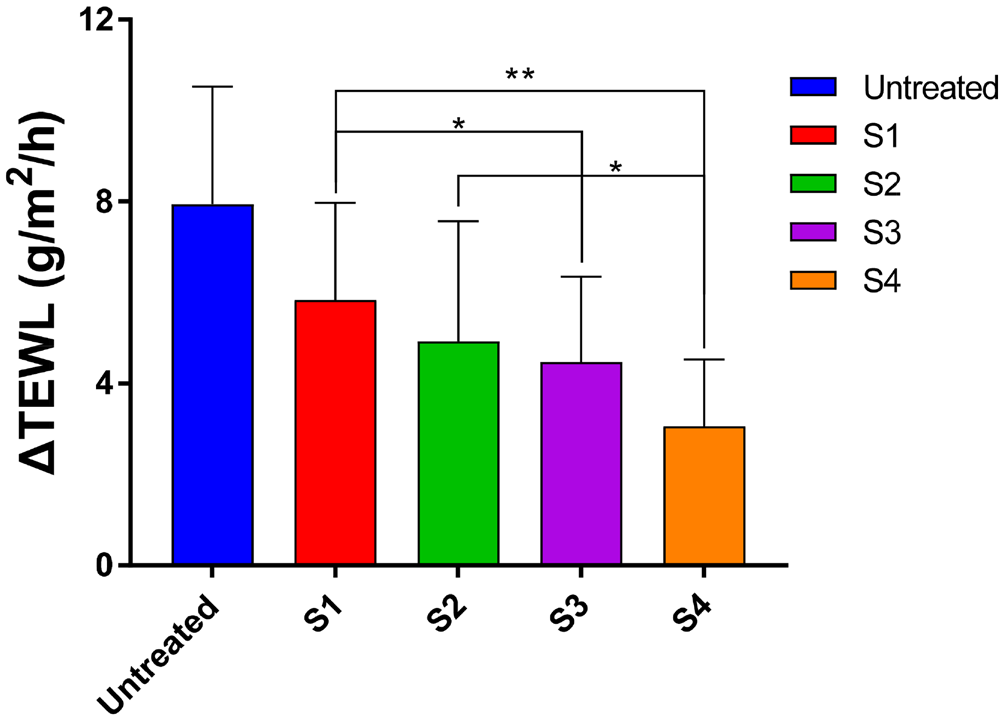
Effects of the test creams on SC cohesion in human participants. ΔTEWL was calculated as the difference between baseline TEWL and TEWL measured after 15 consecutive tape stripping cycles following 4 weeks of application of the test creams (S1, base cream; S2, C18 CER; S3, C16–24 CER; and S4, C24–30 CER). Data were analyzed using a paired t‐test, with statistical significance defined as a *p* < 0.05 (*p < 0.05, **p < 0.01). Results are presented as mean ± SD (*n* = 8).

After the 4‐week application period, 8 of the 21 participants were randomly selected for an assessment of SC cohesion. SC cohesion is defined as the ability of the SC to maintain its structural integrity under physical stress. Following treatment with formulations containing different CER types, the skin was subjected to 15 consecutive tape strips, and SC cohesion was quantified by comparing TEWL values before and after stripping. As shown in Figure 4, all CER‐containing formulations significantly enhanced SC cohesion relative to the vehicle control. Notably, the C24–30 ULC CER group demonstrated a highly significant improvement compared with the C18 CER group, whereas no significant difference was observed between the C18 and C16–24 CER NP groups. A lower ΔTEWL—calculated as the change in TEWL before and after tape stripping—indicates reduced barrier disruption and, consequently, stronger SC integrity.

## 4 Discussion

ULC CERs are recognized as essential contributors to the structural and functional integrity of the skin's permeability barrier, organized in multi‐lamellar lipid layers. Topical application of ULC CERs (C24–30 CER) significantly accelerated barrier recovery, enhanced skin hydration (Figures 2 and 3), and improved SC cohesion (Figure 4). To elucidate the molecular basis for these improvements, alterations in CER content and acyl chain length distribution in the SC were analyzed. Following ULC CER treatment, CER NP species with acyl chains longer than C24 were markedly increased (*p < 0.05*), indicating a strong correlation between elevated ULC CER levels and improved skin barrier function. It is well established that an increase in ULC CERs enhances lipid packing density, particularly by promoting orthorhombic lateral lamellar structure, which reinforces tight lateral packing of the lamellae, thereby increasing structural density and strengthening the permeability barrier. These findings are consistent with previous experimental and lipidomic studies demonstrating the functional advantages of CERs with extended acyl chain lengths in maintaining barrier homeostasis [17, 18, 19, 23].

Results from this study demonstrate that a mixture of ULC CERs (CER C24–30) is significantly more effective than conventional single‐molecule C18 CERs in enhancing multiple aspects of human skin barrier function. Key parameters affected include the rate of acute barrier recovery, skin hydration, and SC cohesion. Consistent with previously published data [29, 33] CER C16–24 also exerted a significant positive effect on skin barrier parameters relative to CER C18, although the effect was less pronounced than that of C24–30 CERs. Since ULC CERs represent the predominant species in the human SC, comprising more than 80% of total CER content irrespective of subclass, the observed results are likely to be correlated with the in vivo human barrier. We did not observe a significant difference between C16–24 CER and C24–30 CER across all three skin‐barrier parameters, likely due to the relatively high ceramide concentration (0.5%) in the test cream. At this level, C16–24 CER may also deliver substantial improvements in barrier performance. A clearer distinction between the two ceramide groups might emerge at lower concentrations, for example, 0.1%–0.2%.

In pathological skin conditions characterized by impaired barrier function, a consistent reduction in average CER chain length has been observed. The relative abundance of CER subclasses varies depending on the type: for instance, NP, NG (NDS), and NH are markedly reduced, whereas NS and AP are increased. These observations highlight acyl chain length as a critical determinant of barrier integrity, potentially exerting a greater influence than subclass distribution alone. Strategies aimed at restoring or maintaining long‐chain CERs may therefore be more effective in improving barrier function than approaches focused solely on altering subclass composition. Moreover, a well‐documented correlation exists between average CER chain length and TEWL, an indicator of barrier function. Studies on atopic dermatitis and other skin disorders reinforce the association between shorter chain lengths and increased TEWL [20]. Complementary evidence from model membrane systems indicates that increasing CER chain length induces a phase transition from a less ordered hexagonal arrangement to a more tightly packed orthorhombic lipid organization [24, 25, 34]. This structural transition is directly linked to decreased permeability to small molecules, reflecting enhanced barrier performance.

In the present study, synthetic CER NP species containing a standardized t18:0 phytosphingosine base were employed to elucidate the contribution of *N*‐acyl chain length to skin barrier function without introducing variability from base heterogeneity. Recent comprehensive lipidomic analysis [6] identified t18:0 as the most abundant long‐chain base species in the human stratum corneum. It accounts for 26.6% ± 2.4% of total long‐chain bases within the CER NP subclass, whereas the t20:0 and t22:0 species constitute 16.4% ± 2.1% and 19.5% ± 2%, respectively. The natural diversity of long‐chain base lengths, including longer bases such as t20:0 and t22:0, may further influence skin barrier function. Future investigations incorporating a broader spectrum of long‐chain base species would therefore be valuable to fully elucidate the role of long‐chain bases in N‐type phytoceramides in skin barrier function.

Despite the recognized importance of CER chain length, direct comparative studies evaluating the effects of ULC CERs versus shorter‐chain counterparts in human skin have been lacking. The present study addresses this gap by providing the first experimental evidence that topical application of ULC CERs to human skin results in significantly greater improvements in skin barrier function compared with conventional C18 CERs. These findings highlight the critical role of acyl chain length in CER‐mediated barrier improvement and support the rational design of CER‐based formulations for optimized skin barrier restoration.

## 5 Conclusions

This study provides the first in vivo human evidence that ULC ceramides (C24–30) enhance skin barrier function more effectively than shorter‐chain CERs. Treatment with a ULC ceramide cream accelerated barrier recovery, improved stratum corneum cohesion, and increased hydration, accompanied by elevated C24–C26 CER NP species within the skin barrier. These findings demonstrate that acyl‐chain length is a key structural factor governing lipid organization and barrier integrity. Because ULC ceramide species predominate in the human stratum corneum, the use of longer‐chain ceramides offers a physiologically relevant approach to restoring barrier integrity and maintaining epidermal homeostasis.

## Author Contributions

Do‐Hyeon Gwon performed liquid chromatography – high mass spectrometry (LC – HRMS) and conducted data analysis and interpretation. Hyun Kyung Choi carried out the human study and processed the human data under the supervision of Sung Kyu Hong and Chang Seo Park drafted the manuscript. Jin Wook Kim prepared the test creams and contributed to the design of the human study. Eun Ok Lee produced the ULC ceramide and formulated the corresponding test cream. Kwang‐Hyeon Liu supervised the overall project, contributed to data interpretation, and assisted in manuscript preparation. All authors have read and approved the final version of the manuscript.

## Funding

This work was supported by Korea Health Industry Development Institute (RS‐2023‐KH141293). Ministry of Health & Welfare (MOHW), Republic of Korea (RS‐2025‐02263556).

## Ethics Statement

All procedures involving human participants were conducted in accordance with the ethical standards of the institutional review board and the 1964 Declaration of Helsinki. Written informed consent was obtained from all participants (IRB approval no. DUIRB‐202403‐08).

## Conflicts of Interest

The authors declare no conflicts of interest.

## Data Availability

Research data are not shared.
